# 1339. Impact of COVID-19 Pandemic on Activity of Other Respiratory Viral Pathogen and Norovirus

**DOI:** 10.1093/ofid/ofab466.1531

**Published:** 2021-12-04

**Authors:** Lauren DiBiase, Emily Sickbert-Bennett, David J Weber, David J Weber, Melissa B Miller

**Affiliations:** 1 UNC Health Care, Chapel Hill, NC; 2 University of North Carolina, Chapel Hill, NC; 3 University of North Carolina School of Medicine, Chapel Hill, NC

## Abstract

**Background:**

The COVID-19 pandemic led to the implementation of several strategies (e.g., masking, physical distancing, daycare/school and business closures, hand hygiene, surface disinfection) intended to mitigate the spread of disease in the community. Our objective was to evaluate the impact of these strategies on the activity of respiratory viral pathogens (other than SARS-CoV-2) and norovirus.

**Methods:**

At University of North Carolina (UNC) Hospitals, we compared the percent positivity for respiratory viral pathogens and norovirus by calendar year for 2014-2019 and the first three months of 2020 to the percent positivity in the subsequent months of 2020 and the first quarter of 2021. Patients were included in the study if they had a positive specimen obtained in a clinic, ED or as an inpatient. Three molecular tests were used to detect these viruses: adenoviruses, endemic coronaviruses (OC43, 229E, NL63, HKU1), influenza A (subtypes H3, H1, H1N1pdm), influenza B, metapneumovirus (MPV), parainfluenza viruses 1-4 (PIV), rhinovirus and/or enterovirus (RhV/EV), and respiratory syncytial virus (RSV). Two molecular tests were used to detect norovirus. We calculated point prevalence rates with 95% confidence intervals to assess statistical differences in percent positivity.

**Results:**

There was a statistically significant decline in percent positivity for endemic coronaviruses, influenza, MPV, PIV, RSV and norovirus during the time-periods after March 2020 when compared to all other time-periods (Figure). RhV/EV, followed by adenovirus were the most prevalent types of respiratory viruses circulating during height of COVID-19. There was a statistically significant decline seen in RhV/EV in April-Dec 2020, but activity increased in 2021. There was no difference seen in adenovirus activity across time-periods.

Percent Positivity of Respiratory Viral Pathogens and Norovirus by Time Period

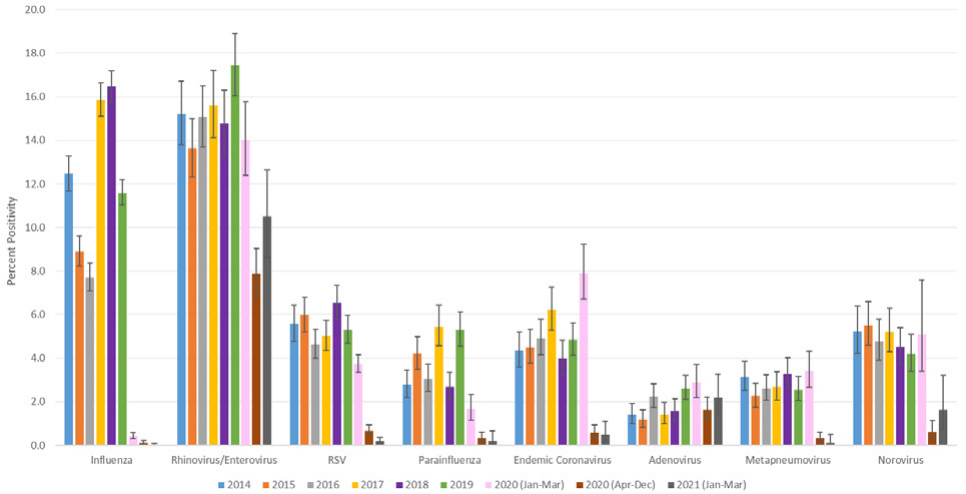

**Conclusion:**

Our study demonstrated statistically significant decreases in the percent positivity of several respiratory viral pathogens, as well as norovirus, during the time-period of high community prevalence of SARS-CoV-2. Strategies put in place to mitigate SARS-CoV-2 transmission likely contributed to these differences. Non-enveloped viruses like rhinovirus and adenoviruses may have been less impacted by these strategies since they are more resistant to disinfection.

**Disclosures:**

**David J. Weber, MD, MPH**, **PDI** (Consultant) **Melissa B. Miller, PhD, D(ABMM), F(AAM**), **Abbott Molecular** (Grant/Research Support)**Agena Bioscience** (Consultant)**ArcBio** (Grant/Research Support)**Cepheid** (Consultant)**Luminex Molecular Diagnostics** (Consultant)**QIAGEN** (Consultant)**Sherlock Biosciences** (Consultant)**Talis Biomedical** (Consultant)**Werfen** (Consultant)

